# Surgeons' workload assessment during indocyanine-assisted deep endometriosis surgery using the surgery task load index: The impact of the learning curve

**DOI:** 10.3389/fsurg.2022.982922

**Published:** 2022-09-05

**Authors:** Emanuela Spagnolo, Ignacio Cristóbal Quevedo, Sara Gortázar de las Casas, Ana López Carrasco, Maria Carbonell López, Isabel Pascual Migueláñez, Alicia Hernández Gutiérrez

**Affiliations:** ^1^ Department of Gynecology, La Paz University Hospital, Madrid, Spain; ^2^ Research Institute, IdiPaz University Hospital, Madrid, Spain; ^3^ Department of General Surgery and Digestive System, La Paz University Hospital, Madrid, Spain

**Keywords:** endometriosis, ICG = indocyanine green, ureter, workload, surg-TLX

## Abstract

**Objective:**

Assess the surgeons' workload during deep endometriosis surgery after ureteral ICG

**Design:**

Prospective, consecutive, comparative, single-center study

**Population:**

41 patients enrolled to deep endometriosis surgery with ureteral ICG from January 2019 to July 2021 at La Paz University Hospital

**Methods:**

Patients were divided into 2 groups: patients operated during the learning curve of ureteral ICG instillation and patients operated after the technique was implemented and routinely performed. After surgery, the SURG-TLX form was completed by the surgeons. We evaluated whether a workload reduction occurred.

**Main outcomes measures:**

Surgeon's workload was measured using the SURG-TLX form, obtaining the total workload and 6 different dimensions (distractions, temporal demands, task complexity, mental demands, situational stress and physical demands)

**Results:**

A significant positive correlation was found between surgical complexity and situational stress (*p* = 0.04). Mental demands (*p* = 0.021), physical demands (*p* = 0.03), and total workload (*p* = 0.025) were significantly lower when the technique was routinely performed. The mental demand, physical demands, and total workload perceived by the surgeons at the beginning of the implementation was higher (68 [39–72], 27 [11–46.5], 229 [163–240], respectively) than in the latter ones (40 [9–63], 11.5 [0–32.8], 152 [133.3–213.8], respectively). Distractions appeared to be higher in the latter surgeries (8.5 [0–27.8]) than in the first surgeries (0 [0–7]; *p* = 0.057).

**Conclusions:**

Ureter ICG instillation prior to DE surgery significantly reduces the mental and physical demands and total workload of the surgeons in DE surgeries after overcoming the learning curve. Distractions appear to increase as surgical stress decreases.

## Introduction

Deep endometriosis (DE) is defined as the presence of endometrial tissue that infiltrates the peritoneum more than 5 mm (1, 2) and it affects between 3%–37% of premenopausal women (1, 3–6). The most common DE locations within the intestinal tract are the rectum and the rectosigmoid junction, with a prevalence of 52.0%–65.7% (3).

In case of large rectal nodules or posterior vaginal nodules (>3 cm), the ureters are often involved, given the inflammatory response frequently spreads, with a distortion of normal anatomy (7, 8). One of the great dangers is that asymptomatic ureteral endometriosis can lead to silent progressive kidney function loss (9, 10).

Ureteral endometriosis is defined as any condition in which endometriosis causes compression or alteration of the regular ureteral anatomy (7). The disease has a prevalence of up to 50% in women with DE (11), and it commonly affects the distal segment, 3–4 cm above the vesico-ureteric junction (12). Furthermore, bilateral involvement can be present in 10%–20% of cases, although is usually unilateral with a left predisposition (13). Ureteral compression is common in patients with ureteral endometriosis, especially in women with parametrial infiltration and a low BMI (14).

DE surgery represents a challenge and can be a stressful event, even in experienced multidisciplinary teams. Distortion of the common anatomy due to large endometriotic nodules can affect the 3 pelvis' compartments, as well as multiple adhesions made by previous surgeries, make the surgical procedure complex and with a high risk of ureteral injuries.

The performance of DE surgery requires a state of attention (ability to “be alert”) and concentration (ability to be aware of an activity or a set of activities for a period of time), that implies a considerable workload (15). Workload is a multifaceted construct, determined by the interaction of the task demands, the circumstances under which the task is performed, and the skills, behaviors, and perceptions of the individual (16, 17).

The introduction of fluorescence-guided surgery, which allows a real-time visualization of certain anatomical structures, could help surgeons to reduce their workload and improve performance. Recently, the intraoperative use of indocyanine green (ICG) has shown potential advantages. In particular, some authors have described the use of ICG as a feasible, safe tool to assess ureteral perfusion during DE surgery (18, 19). Historically, systematic placement of bilateral ureteral stents has been routinely used for DE surgeries (20). Our team has recently reported the use of ICG intraureteral instillation to provide a real-time visual assessment of the ureters during surgery (21).

The aim of this study was to analyze which factors affect the surgeon's workload during DE laparoscopic surgery after the introduction of intraureteral ICG, using the validated Surgery Task Load Index (SURG-TLX) (17).

We also evaluated whether a significant workload reduction occurred during surgeries in the study period due to intraureteral ICG use during DE surgery.

## Materials and methods

We conducted a prospective, single-center, preliminary study, which enrolled consecutive symptomatic women scheduled for laparoscopic surgery for DE during January 2019 and July 2021 at our Endometriosis Unit.

The study was approved by the local Ethics Committee (code number resolution: HULP PI.3592). A written informed consent was given to every women who was eligible for our study by the surgeons who were going to operate them. Also, surgeon's gave their written consent to study their results on the questionnaries.

The inclusion criteria were patients with DE who signed the written informed consent, were of legal age, had no allergy to iodine, and had a disposition to follow-up. Patients were excluded who had any condition that contraindicated ICG use (e.g., liver or kidney function alterations, history of thyrotoxicosis).

All patients underwent a gynecologic examination and an exhaustive pelvic ultrasound scan executed by expert sonographers at our center. In selected cases, magnetic resonance imaging was additionally performed (12). When severe bowel involvement was suspected, colonoscopy or endoanal ultrasound were performed. Finally, a urological computed tomography scan was performed in case of probable severe damage to the urinary tract. After detailed counseling, all the included patients signed an informed consent form for DE surgery and the use of ICG. Demographic data, the severity of pain symptoms (chronic pelvic pain, dysmenorrhea, dyspareunia, dysuria, dyschezia) was assessed through the visual analog scale (22), and surgical data were collected. Postoperative complications were registered employing the Clavien-Dindo score (23).

Surgical complexity was estimated by totaling the individual procedures performed performed in every patient during the surgery (Table 2).

**Figure 1:**
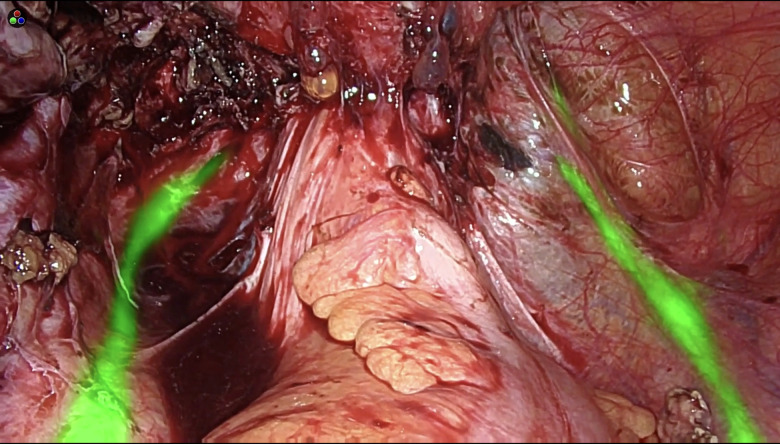
ICG visualization of the ureters during DE laparoscopic surgery.

The technique used for the ureteral assessment by ICG has already been described (21). In the operating theatre, before starting the laparoscopic procedure, a 6 Fr ureteral catheter was introduced into each ureter at cystoscopy, and 7 ml ICG solution (1.25 mg/ml of distilled water) was injected. The catheter was then removed. Using a near-infrared camera (Olympus Medical Systems Europa, Hamburg, Germany or Stryker Iberia S.L., Madrid, Spain), the fluorescent ureters could be visualized during the entire laparoscopic surgery procedure, in overlay mode, without needing to change the camera mode (Figure 1).

All laparoscopic surgeries were performed by two DE-experienced surgeons, who completed the SURG-TLX form (17) at the end of the procedure.

SURG-TLX is a multidimensional surgery-specific workload measure that has been developed to study 6 dimensions, defined as follows:

– Mental demands (How mentally fatiguing was the procedure?)
– Physical demands (How physically fatiguing was the procedure?)
– Temporal demands (How hurried was the pace of the procedure?)
– Task complexity (How complex was the procedure?)
– Situational stress (How anxious did you feel while performing the procedure?)
– Distractions (How distracting was the operating environment?)

A 2-part evaluation is required to complete the SURG-TLX. The first part involves calculating weights of the 6 dimensions following a set of 15 paired comparisons. The dimension with the highest weight is the most important contributing factor for the perceived workload (scores range from 0 to 5).

The second part involves rating 6 bipolar scales, reflecting the individual dimensions on a 20-point Likert scale, anchored between low and high. A workload score for each dimension is then calculated by determining the product of these two numbers. For example, a weight score of 4 and a rating of 15 equate to a workload score of 60 (scores range from 0 to 100). A total workload score (“how demanding was the task?”) is also determined by aggregating the scores from the 6 dimensions (17); the higher it is, the more demanding the task.

We determined which factors affected the surgeon's total workload. Furthermore, we evaluated whether a significant workload reduction occurred during the surgeries in the study period due to the use of ICG imaging for intraoperative assessment of the ureters, dividing two time periods: surgeries performed during the learning curve of ureteral ICG use (group 1) and patients operated when the technique was well implemented and routinely performed in DE complex surgeries (group 2).

### Statistical analysis

Afterward, a database was created to store information on the patients, and the statistical analysis was performed with SPSS statistical analysis tools (SPSS Inc., Chicago, IL, USA). Regarding the continuous variables, we first examined the normality of these variables so they can be described in a manner that best suits the characteristics of each. We described the variables that follow normality as means ± standard deviation, and those that did not do so as medians and interquartile ranges. We expressed the categorical variables as absolute numbers and percentages. Correlations for the continuous variables were performed using a non-parametrical statistical analysis, *Spearman's rho*. For the difference of medians for categorical variables, a Mann–Whitney U test was performed. It was considered an 80% of statistical power and 5% of probability of error (*p*) in all statistical analysis.

## Results

A total of 41 patients were enrolled, assigned to 2 inclusion periods: the first period included 19 (46.3%) patients in 2019 during the learning curve of ureteral ICG instillation; the second period included 22 (53.7%) patients in 2020–2021, when ureteral ICG instillation was routinely performed. Two experienced surgeons, with a mean age of 39.8 years, completed the SURG-TLX form at the end of each procedure, with a 100% response rate.

The demographic and presurgical clinical data of all women are summarized in Table 1, all had more than 1 lesion, and the surgical procedures performed are reported in Table 2.

**Table 1:** Women's’ demographic and clinical data.

Age (mean, SD)	38.46±4.78
Body mass index (mean, SD)	26.11±5.34
Obstetric record (*n*, %):	
Previous cesarean delivery	9 (22%)
Previous vaginal birth	7 (17.1%)
-None	25 (61%)
Previous surgery for endometriosis (*n*, %)	20 (48.7%)
Previous surgery not for endometriosis (*n*, %)	16 (39%)
Preoperative pain symptoms assessed by visual analog scale (mean, SD)	
Dysmenorrhea (0–10)	6 ± 3.94
Dyspareunia (0–10)	3.71 ± 3.86
Dysuria (0–10)	1.05 ± 2.3
Chronic pelvic pain (0–10)	4.02 ± 3.85
Other deep endometriosis symptoms (*n*, %)	
Constipation	13 (31.71%)
Some level of urinary obstruction	9 (21.9%)
Preoperative medical treatment (*n*, %)	
Combined oral contraceptives	19 (23.17%)
-Oral progestins	13 (15.85%)
Hormonal intrauterine device	11 (13.41%)
Gonadotropin-releasing hormone agonist	11 (13.41%)
Ultrasound findings (*n*, %):	
Cystic endometriosis	27 (65.9%)
Uterosacral ligament	18 (43.9%)
Rectovaginal septum	10 (24.4%)
Torus	9 (21.95%)
Bowel’s muscular layer	9 (21.95%)
Magnetic resonance imaging findings (*n*, %) performed in 23 patients:	
Cystic lesions	20 (86.96%)
Uterosacral ligament	6 (26.08%)
Rectovaginal septum	4 (17.4%)
Torus	8 (34.78%)
Bowel’s muscular layer	3 (13.04%)

**Table 2:** Surgical procedures.

Hysterectomy (*n*, %)	23 (56.1%)
Ovarian cystectomy (*n*, %)	19 (46.34%)
Salpingectomy (*n*, %)	31 (75.61%)
Adhesiolysis (*n*, %)	35 (85.4%)
Associate endometriosis lesion (*n*, %)	
Uterosacral ligament	12 (29.3%)
Recto-vaginal septum	28 (68.3%)
Lateral parametrium	2 (4.8%)
Bowel surgery (*n*, %)	
Shaving	16 (39%)
Discoid resection	5 (12.2%)
Segmental resection	6 (14.63%)
Urinary tract surgery (*n*, %)	
Ureterolysis	23 (56.1%)
Ureteral nodule excision	3 (7.3%)
Ureteral reimplantation	2 (4.9%)
Partial bladder cystectomy	1 (2.4%)
Bladder nodule resection	3 (7.3%)
(without vesical opening)	

Laparoscopic surgery was performed in 39 (95.1%) patients. Conversion to laparotomy was necessary in 2 (4.9%) due to multiple adhesions.

Two (4.9%) patients underwent segmental rectal resection that required protective ileostomy due to ultralow anastomosis (less than 5 cm from the anal verge) (5, 24). Regarding bowel nodules, mean nodule size was 23.7 ± 8.3 mm, with a mean length of the intestinal resection of 7 ± 5.6 cm, when discoid resection or intestinal segmentary resection were performed. Mean operative time in group 1 was 317.1 ± 78 min, and 254.3 ± 69.3 min in group 2, showing a statistically significant difference of *p* = 0.01. The mean hospital stay throughout the study was 5.2 ± 2.7 days, without showing any differences between the groups (*p* = 0.15).

Regarding the SURG-TLX form, we found a strong positive correlation (r_39 _= 0.3, *p* = 0.04) between the number of procedures performed during surgery (adding complexity to the surgery) and an increase in situational stress.

No statistically significant association was found between mental demands, temporal demands, physical demands, task complexity, situational stress, distractions or total workload and patients' body mass index (BMI), the bowel nodule removal technique, or the number of previous surgeries.

Subsequently, we compared the SURG-TLX form between the 2 inclusion periods: the DE surgeries performed in group 1 and group 2 DE surgeries. These comparisons are shown in Table 3.

**Table 3:** Comparison of the SURG-TLX forms between both groups of surgeries.

	Mental demands	Physical demands	Temporal demands	Task complexity	Situational stress	Distractions	Total workload
2019 surgeries median (range)	68 (39–72)	27 (11–46.5)	26 (22–34.5)	52 (11–72)	30 (11.5–52.5)	0 (0–7)	229 (163–240)
2020–2021 surgeries median (range)	40 (9–63)	11.5 (0–32.8)	34.5 (12.5–44)	44 (28.5–66.3)	18 (14–38.3)	8.5 (0–27.8)	152 (133.3–213.8)
Total surgeries median (range)	54 (26–71)	17 (5.5–35)	28 (15–44)	48 (23–72)	18 (12–43)	4 (0–22)	204 (142,5–236,5)
Mann–Whitney test (*p*)	0.021	0.03	0.844	0.948	0.313	0.057	0.025

Showing the median (interquartile range) of the 6 workload items and the comparison of both groups.

**Figure 2:**
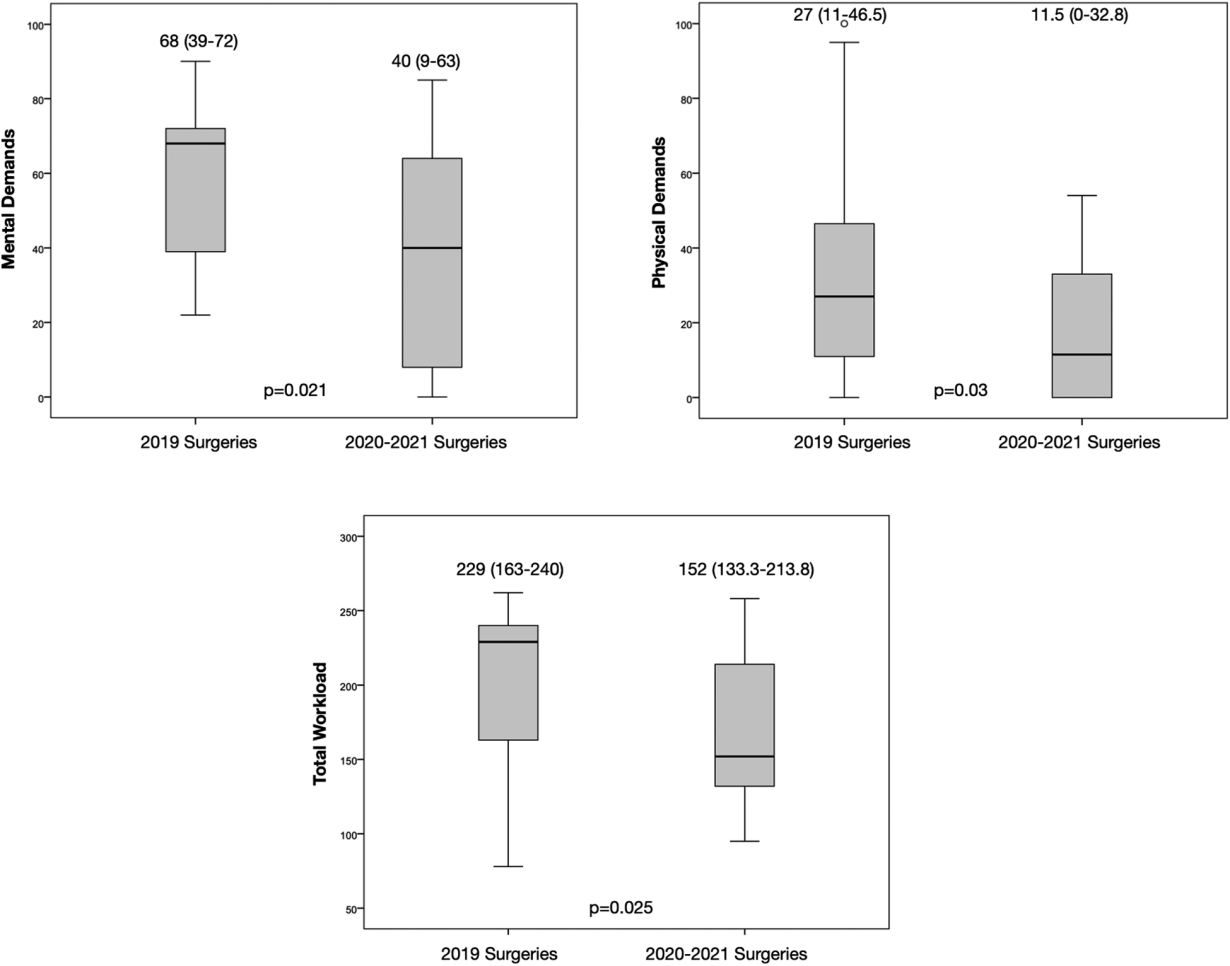
Boxplot comparing mental and physical demands and total workload between the two surgery groups.

We found a significant difference in terms of mental demands (*p* = 0.021), physical demands (*p* = 0.030), and total workload (*p* = 0.025), comparing both studied groups, as shown in Table 3. The mental demands, physical demands, and total workload perceived by the surgeons in group 1 was higher (68 [39–72], 27 [11–46.5], and 229 [163–240], respectively) than in group 2 (40 [9–63], 11.5 [0–32.8], and 152 [133.3–213.8], respectively), as shown in Figure 2.

A trend toward significance difference was shown in distractions (*p* = 0.057), with the group 1 surgeries (0 [0–7]) showing a lower level of distractions than the later ones (8.5 [0–27.8]), as shown in Table 3.

On the other hand, no statistically significant differences were observed when comparing both groups of surgeries in terms of temporal demands (26 [22–34.5], 34.5 [12.5–44]; *p* = 0.844), task complexity (52 [11–72], 44 [28.5–66.3]; *p* = 0.984), and situational stress (30 [11.5–52.5], 18 [14–38.3]; *p* = 0.313).

## Discussion

### Main findings

Mental, physical demands and the total workload decrease after overcoming the learning curve of Ureter ICG instillation technique applied to DE surgery, as measured by the multidimensional SURG-TLX form, although it has been observed that distractions may increase as the ICG ureteral technique becomes established.

### Strengths and limitations

Regarding the limitations of our study, our sample size was limited. This study could be considered a pilot study to assess the benefits of the ICG in DE surgeries. Furthermore, SURG-TLX does not include how much the surgeon rested the night before, as a factor that may involve the surgeon's technique. A surgeon who is more comfortable with this technique could confer its benefits to our patients, and on a larger scale, to our hospital, given that fewer complications can lower rates of DE and shorter hospital stays can lead to an economic benefit. Additionally, it would have been valuable to be able to compare the workload outcome in two different groups of patients, one with the ICG technique and one without.

However, indocyanine is already widely used in gynecology (25). It will be interesting to analyze larger series with a longer follow up to show a clear benefit of this technique in DE surgery. On the other hand, the strength of our study is that, to our knowledge, it is the first study to apply the SURG-TLX to evaluate the improvement in the surgeon's comfort during DE surgery.

### Interpretation

In the operating room, adverse events are often generated by a combination of factors, such as the number of scheduled cases, pressure to perform complex tasks, and conflicting priorities, which can lead to added mental tension, stress, and poor teamwork (26, 27). At our center, we started using ureteral ICG-guided surgical procedures in patients with DE to reduce surgical complexity, because the pelvic anatomy can be deeply distorted. ICG helps to visualize the ureters during surgery (21), preventing, or at least limiting, the possible iatrogenic damage. It has to be mentioned, that in our study we had a similar percentage of conversion to laparotomy than in the literature (28).

The use of ICG imaging technology in DE surgery has been described as safe and feasible in recent years (18, 29).

According to mental ergonomics, with time, the novelty of a new technique will become routine, and experienced surgeons feel that they perform better with better physical ergonomics (30). Furthermore, this technique helps with one of the most critical steps of pelvic surgery: to locate the ureter (31), which can lead to a rapid ureter visualization. This can avoid the surgical dissection of the anatomical spaces trying to prevent any iatrogenic damage (32). It is not surprising that the introduction of this technique (21) helps surgeons during surgery and reduces their workload. DE surgery can be unpredictable due to an altered anatomy, contributing to a more complex surgery that leads to an increase in the situational stress perceived by the surgeon. With the advancement of technology in surgery, we expect that the surgeons' stress could decrease in the future, even though some level of stress will always be experienced because these surgeries will continue to be complex.

We found a strong positive correlation between the complexity of the surgeries and the situational stress. A more complex surgery led the surgeon to perceive more situational stress. In 2018, Lowndes et al. reported that when procedural difficulty is greater than expected, physical and mental demand as well as situational stress increase significantly (33). DE lesions do not always correspond to presurgical diagnostic imaging, sometimes leading to unpredictable surgeries, which as observed in our study, leads to an increase in surgeons' stress. Given that our surgeries were always videotaped and performed by the same experienced surgeons, no ego-threat affected the situational stress dimension, as has been demonstrated by others (17).

In our study, mental and physical demands and total workload were significantly lower in the second period. As Bin Zheng et al. assessed, practice develops automaticity, which reduces the mental workload. When automaticity occurs, movements are performed consistently and efficiently without requiring many mental resources (34), probably causing less fatigue. This allows surgeons to have sufficient mental resources to attend to a secondary task. The introduction of new techniques in the operating room can increase the surgeon's disturbance without showing any ergonomic advantage (35). This increase was reinforced in our study, given that the introduction of our technique generated a higher workload than during the second period.

Our 3-year study has followed our surgical experience with ureter ICG instillation, showing that in group 2 surgeries, distractions were significantly higher than in group 1 surgeries. This effect has been reported in previous studies, because teams are particularly vulnerable to distractions when stress and workload are low, highlighting the need to remain ever-vigilant throughout a procedure (26). Although some distractions might be inevitable, others, particularly during tasks that require total attention, should be proactively controlled because they can induce error and have negative consequences on patient safety (36). To some extent, distractions in the operating room can be managed through “systematic” interventions, such as the creation of regular preoperative briefings (37) or the introduction of standard operating protocols (including, for example the concept of the “sterile operating room,” where no unnecessary conversations occur at safety-critical points of a procedure). At other times, the successful management of these distractions might rely upon strong surgical leadership and team-member commitment. In fact, the ability to effectively manage errors and unexpected events is a “marker of surgical excellence” (26).

In our study, the BMI of our patients did not affect the surgeons' workload in any of the individual items or even in the total workload. These results are aligned with Lowndes et al., who did not find any correlation between BMI and total workload, but which differ from previous literature (33). Patients with higher BMI are known to be more challenging, given that more time is typically needed to perform the surgery (38, 39). We could not find any differences in the operating time between the two groups, probably because of the small cohort (18). On the other hand, longer surgeries are associated with greater amounts of perceived cognitive load for the team, including higher perceived mental and physical demand, task complexity, degree of difficulty, distractions, and total cognitive load (40).

Although the nodule size is important to determine the surgical technique (41, 42), no significant differences were observed between the nodule size, the 6 items, and the total workload. This absence of correlation could be explained by the protocol implemented in our center, to help with “decision-making” when it comes to bowel endometriosis. Stress can be perceived by the surgeon when deciding between shaving, discoid, and segmental resection. This stress has been addressed in our center, by establishing that multiple nodules or nodules >3 cm routinely undergo segmental resection.

We found no significant differences with patients' previous surgeries, DE-related or not. The meta-analysis by Ten Broek et al. concluded that because complications of postoperative adhesion formation are frequent and have a considerable negative effect on patients' health (43), later surgeries might be more challenging than the first ones, and the postoperative recovery time can be longer (44). Neither of these outcomes were observed in our study. Furthermore, patients with endometriosis are young, and the more DE surgeries they undergo, the fewer positive outcomes can be expected of their ovarian reserve (45).

Finally, surgeons could manage a higher workload, given that each individual item studied barely reached 50% of the scale; when combined, however, the total workload reached to nearly two-thirds of the total workload the surgeons could handle. As previously mentioned, we should not view the workload a surgeon can handle as a unique item, but it must rather be conceived as a complex multidimensional element (16, 17). When an imbalance is present such that cognitive load is excessive, surgical team members' ability to adapt to changing work demands is diminished, and their likelihood of committing cognitive errors is enhanced (40).

## Conclusion

Surgeon's workload decreases after overcoming the learning curve of ureteral ICG instillation technique, as measured by the SURG-TLX form, although it has been observed that distractions can increase as the technique becomes established.

## Data Availability

The raw data supporting the conclusions of this article will be made available by the authors, without undue reservation.
